# Editorial: The protein kinase GSK3 in neurobiological functions and neuronal disorders

**DOI:** 10.3389/fnmol.2023.1155905

**Published:** 2023-02-27

**Authors:** Florian Plattner, Shin-ichi Hisanaga, Raymond Chuen-Chung Chang, Anna Matynia

**Affiliations:** ^1^Neuro-Research Strategies, Houston, TX, United States; ^2^Department of Biological Sciences, Tokyo Metropolitan University, Hachioji, Japan; ^3^Laboratory of Neurodegenerative Diseases, LKS Faculty of Medicine, School of Biomedical Sciences, The University of Hong Kong, Pokfulam, Hong Kong SAR, China; ^4^Jules Stein Eye Institute, University of California, Los Angeles, Los Angeles, CA, United States

**Keywords:** glycogen synthase kinase 3, protein kinase, pharmacological inhibitor, lithium, neurodegenerative diseases, bipolar disorder

Since its initial discovery more than 40 years ago, a series of significant discoveries and innovative research established a central role for glycogen synthase kinase 3 (GSK3) in many critical cellular processes, including gene expression, metabolism, cellular transport, cell proliferation and apoptosis. GSK3 is a highly conserved serine/threonine protein kinase that is present in all eukaryotes. GSK3 has a broad substrate range and is ubiquitously expressed. Interestingly, GSK3 is present at high levels in brain and specifically phosphorylates numerous neuronal proteins implicating it in a variety of neurobiological functions such as axonal transport, neuronal development, neuronal polarization as well as in the regulation of synaptic plasticity and memory formation (e.g., Hernández et al., 2002; Hooper et al., 2007). Consistently, dysregulation of GSK3 has been associated with neuronal disorders, including Alzheimer's disease, bipolar disorder, schizophrenia, Fragile X Syndrome and Parkinson's disease. This central role in neurobiological functions and neuronal disorders has made GSK3 an attractive therapeutic target and sparked major drug development efforts.

This Frontiers Research Topic features original research articles and reviews that provide new insights on neurobiological functions of GSK3 and an update on the current state of the GSK3 research field with a special emphasis on GSK3 inhibitors in pre-clinical research and their potential therapeutic application. We assembled this Topic with the aim to provide a contemporary reference point for GSK3 research and to stimulate future progress in our understanding of this complex field.

Over the years, an enduring and far-reaching effort to develop specific GSK3 inhibitors has been driven by a number of critical discoveries. In contrast to most protein kinases, GSK3 can be active in its basal state and is inhibited in response to various physiological stimuli. Consistently, dysregulation and hyperactivation of GSK3 have been associated with numerous, diverse pathophysiological changes, which continuously prompted the demand for diverse, selective, and potent GSK3 inhibitors. The first GSK3 inhibitor discovered, was the cation lithium (Klein and Melton, 1996; Stambolic et al., 1996). Lithium is a commonly prescribed mood stabilizer, for which the mechanism of action is still not known. In this Topic, Chatterjee and Beaulieu review the evidence that links GSK3 inhibition to the therapeutic effects of lithium on mood. This review provides an overview of GSK3 biological functions and substrates that have been implicated in the effects of lithium with a focus on the transcription factor cAMP response element-binding protein (CREB), the RNA-binding protein FXR1, kinesin subunits, and the cytoskeletal regulator CRMP2. Indeed, understanding the mechanistic link between GSK3 inhibition and the therapeutic effects of lithium may prove critical to identify novel, more specific drug targets and to provide a rationale for the inhibitory action of ketamine and other antipsychotics toward GSK3.

Following up on the recent advances in the development of pharmacological GSK3 inhibitors and their application in pre-clinical and clinical studies of neuronal disorders, is the next review in this Topic. Arciniegas Ruiz and Eldar-Finkelman present the current spectrum of GSK3 inhibitors of diverse chemotypes and inhibition modes, and summarize the use of these GSK3 inhibitors in clinical trials and *in vivo* animal models of CNS disorders ranging from mood and behavior disorders, autism and cognitive disabilities, to neurodegeneration, brain injury and pain. Despite there being currently no specific GSK3 inhibitors on the market, recent advances are encouraging and hopefully will lead to the development of GSK3 inhibitors suitable for clinical use.

The importance of the development of novel GSK3 inhibitors for the study of neurobiological functions and pathophysiological processes is further exemplified by the original articles from Westmark et al., Di Re et al., and Lee et al. in this Topic.

Taking advantage of an animal model for Fragile X Syndrome, a genetic condition causing intellectual disability, Westmark et al. investigated the effects of two different pharmacological GSK3 inhibitors on disease-associated behavioral changes *in vivo* as well as on biochemical and cytological read-outs in neuronal cultures. These experiments revealed an intriguing interplay between GSK3 activity and the expression of amyloid-beta precursor protein (APP), a molecule that has been critically implicated in Alzheimer's disease amongst other CNS disorders. This study also uncovered differential effects of the two tested GSK3 inhibitors on some of the read-outs, highlighting common complications encountered with pharmacological inhibitors. Functional differences between inhibitors may be due to a variety of factors, such as different specificity, off-target effects and altered bioavailability. Many commonly used inhibitors are directed toward the ATP binding site of a specific kinase. However, as ATP binding domains exhibit a high degree of structural homology between kinases, confounding side effects due to cross-reactivity are a common problem of this type of inhibitors.

Di Re et al. examined the effect of pharmacological inhibition of GSK3 and of its inhibitory upstream regulator, Akt, on the functionality of the axon initial segment (AIS) that is required for neuronal firing. GSK3 inhibition induced no major changes, whereas Akt inhibition resulted in increased excitability in primary hippocampal neurons and altered subcellular localization of βIV spectrin.

Lee et al. investigated the effect of pharmacological GSK3 inhibition on synaptic plasticity and memory formation *in vivo* in this Topic. Systemic administration of a selective GSK-3 inhibitor, CT99021, reversibly blocked NMDAR-dependent long-term depression (LTD) in the CA1 region of the hippocampus and facilitated learning in the Morris water maze (MWM) and T-maze. These data suggest that GSK3 may be involved in the fine-tuning of spatial memory acquisition and recall.

The GSK3 inhibitor, CT99021, while selective for GSK3, does not discriminate between the two GSK3 isozymes that are generated from distinct genes. The two GSK3 isoforms, termed α and β, share high levels of homology, feature near identical substrate specificity and hence may have largely a common substrate range. Nevertheless, it has also become evident that there are GSK3 isoform-specific substrates and functions. Indeed, GSK3 isoform-inhibitors have been developed. In addition, there might also be functional interplay between the two GSK3 isoforms, which adds an additional layer of complexity to the analysis of GSK3 functions in neurobiological and pathophysiological processes.

In order to gain insight on GSK3 isoform-specific roles in synaptic plasticity, Amini et al. evaluated long-term potentiation (LTP) in CA3-CA1 of the hippocampus from conditional GSK3α and GSK3β knockout mice in this Topic. Deletion of GSK3α in CA1 pyramidal neurons resulted in facilitation of CA3-CA1 LTP, whereas GSK3β deletion had no effect on LTP. Basal synaptic properties were not affected by either GSK3 deletion. These results support the notion that GSK3α plays a specific role in regulating CA1 LTP and adds a further example of diverging functions between GSK3 isoforms.

But as occurs so often in research and is highlighted by the articles in the Topic, finding a novel answer raises a myriad of new, unsolved questions. In fact, despite a considerable research effort the neurobiological and pathophysiological roles of GSK3 remain still poorly understood. The complex biology of GSK3, including two isoforms, diverse regulatory mechanisms, countless substrates and its involvement in many different cellular functions, makes this a challenging research field and progress is hard to come by. Over the last few years, progress has been further hampered due to a shift in research focus away from investigating the mechanistic basis of physiological and pathophysiological processes as well as to changing funding priorities by sponsoring bodies.

As we reflect on the past 40 years, there have been tremendous advances that single out GSK3 as a relevant, druggable target. Future research will unequivocally progress our mechanistic understanding of GSK3 functions and promote the development of GSK3-based therapeutic applications.

## Author contributions

All authors listed have made substantial, direct, and intellectual contribution to the work and approved it for publication.
